# Reflective oxygen saturation monitoring at hypothenar and its validation by human hypoxia experiment

**DOI:** 10.1186/s12938-015-0071-z

**Published:** 2015-08-05

**Authors:** Tao Guo, Zhengtao Cao, Zhengbo Zhang, Deyu Li, Mengsun Yu

**Affiliations:** School of Biological Science and Medical Engineering, Beihang University, Beijing, China; China Astronaut Research & Training Center, Beijing, China; Research Center of Aviation Medicine Engineering, Institute of Aviation Medicine, Beijing, China; Department of Biomedical Engineering, Chinese PLA (People’s Liberation Army) General Hospital, Beijing, China

## Abstract

**Background:**

Pulse oxygen saturation (SpO_2_) is an important parameter for healthcare, and wearable sensors and systems for SpO_2_ monitoring have become increasingly popular. The aim of this paper is to develop a novel SpO_2_ monitoring system, which detects photoplethysmographic (PPG) signals at hypothenar with a reflection-mode sensor embedded into a glove.

**Methods:**

A special photo-detector section was designed with two photodiodes arranged symmetrically to the red and infrared light-emitting diodes (LED) to enhance the signal quality. The reflective sensor was placed in a soft silicon substrate sewn in a glove to fit the surface of the hypothenar. To lower the power consumption, the LED driving current was reduced and energy-efficient electronic components were applied. The performance for PPG signal detection and SpO_2_ monitoring was evaluated by human hypoxia experiments. Accelerometer-based adaptive noise cancellation (ANC) methods applying the least mean squares (LMS) and recursive least squares (RLS) algorithms were studied to suppress motion artifact.

**Results:**

A total of 20 subjects participated in the hypoxia experiment. The degree of comfort for wearing this system was accepted by them. The PPG signals were detected effectively at SpO_2_ levels from about 100–70%. The experiment validated the accuracy of the system was 2.34%, compared to the invasive measurements. Both the LMS and RLS algorithms improved the performance during motion. The total current consumed by the system was only 8 mA.

**Conclusions:**

It is feasible to detect PPG signal and monitor SpO_2_ at the location of hypothenar. This novel system can achieve reliable SpO_2_ measurements at different SpO_2_ levels and on different individuals. The system is light-weighted, easy to wear and power-saving. It has the potential to be a solution for wearable monitoring, although more work should be conducted to improve the motion-resistant performance significantly.

## Background

As claimed by American Heart Association, cardiovascular diseases have been the leading cause of death [1]. It is very critical to monitor patient’s physiological signals [such as pulse oxygen saturation (SpO_2_)] continuously and noninvasively before diagnosis or treatment of cardiovascular diseases. However, traditional monitoring methods are usually conducted in a short time window, which are likely to lose signals of transient events that may be of profound prognostic or therapeutic importance. To solve this problem, some wearable health monitoring devices (WHMDs) have been developed to monitor specific signals continuously and automatically [2].

In recent years, pulse oximetry has been extensively used in WHMDs to measure SpO_2_ and heart rate noninvasively [3]. It was invented in the 1970s [4] and has been continuously improved since then, which is based on the detection of subcutaneous blood perfusion by irradiating light into the skin. The subdermal blood volume changes due to arterial pulsations modify the absorption, reflection or scattering of the incident light. Consequently, the fluctuation of resultant reflective/transmittal light intensity, i.e. photoplethysmograph (PPG), can indicate heart rate and other hemodynamic parameters that are related to local blood volume changes. Through the different spectral absorption coefficients of oxygenated and non-oxygenated blood, SpO_2_ can also be measured by using multiple wavelengths (pulse oximetry) [5].

Commercial PPG sensors usually work in transmission mode that needs the incident light sent by their emitters, red and infrared light-emitting diodes (LED), to penetrate the tissue at measuring sites to reach their detectors, photodiodes (PD). This work mode has its limitations—it can only be used in body regions which are not opaque to the light, such as the fingertip and earlobe. Unfortunately, both of these two regions are not ideal sensor locations for continuous monitoring. The former is occupied in most daily activities and the latter usually requires a clip which may cause discomfort during long time measurements [6]. While a reflective PPG sensor uses the back-scattered or reflected light to measure, it can be chosen to overcome those limitations. It allows the LED and PD to be mounted next to each other on the same planar surface and thus enables monitoring SpO_2_ at multiple locations of the body where transmission measurements are not practical.

Based on the reflective technology, a number of wearable pulse oximeters (WPOs) have been developed during the past decade. One challenge to WPO design is how to balance comfortable wearing and reliable attachment. Patterson et al. [7] developed a multichannel PPG sensor placing around the ear, which needed a wired connection with processing module carried somewhere else on the body and thus is not convenient for wearing. This problem also exists in the design of Buschmann et al. [8], which embedded a SpO_2_ sensor inside an ear mould for measurement in the external auditory canal. Mendelson et al. [9] reported a more integrated solution that assembled the senor, power source and data handling in one module fixed on the forehead by a bandage around the head. To ensure good signal quality and reliable sensor attachment, the bandage should not be tied too loose, limiting the wearing comfort. Haahr et al. [10] devised a WPO in the form of an electronic patch, which contains an adhesive material that can unite it to the body. This patch is a single unit without wires and does not limit movements. However, confined to a small size, it only carries a coin size battery and thereby its duration of wireless data transmission is restricted.

This study presents a new integrated solution for WPO, which incorporated the sensor, power supply, data processing and wireless transmitting into a glove, realizing SpO_2_ measurement at the hypothenar. By this form, the sensor can be attached reliably during daily activities without limiting the movements of hand and with little discomfort for long-time wearing. Methods to decrease power consumption, such as decreasing the LED driving current, were applied in this system to achieve a long period of measurement with wireless communication. The device was evaluated under various levels of hypoxia and the results demonstrated that determination of oxygen saturation at hypothenar by this system is feasible. However, monitoring SpO_2_ at this location is vulnerable to motion interference. Therefore, accelerometer-based adaptive noise cancellation (ANC) methods using least mean squares (LMS) and recursive least squares (RLS) algorithms were adopted and evaluated on their effectiveness of suppressing motion artifacts.

## Methods

### Configuration of the system

**Fig. 1:**
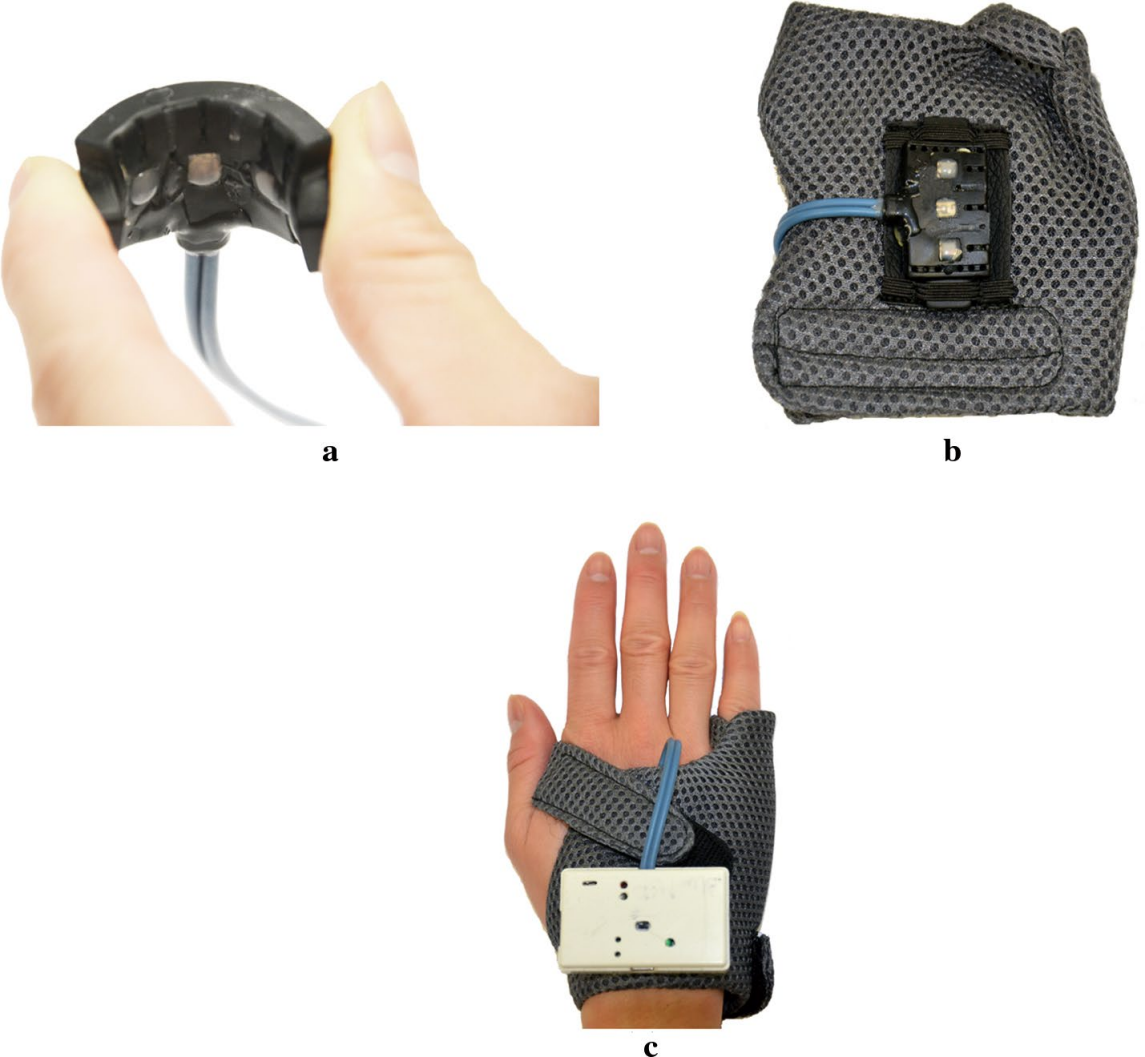
Overview of the system. **a** The sensor node. **b** *Inside view* of the glove and position of the sensor. **c** Wearing appearance of the system.

The system is composed of three parts: a reflective PPG sensor, a data processing package, and a glove (Fig. 1). The LED and PD of the sensor are encapsulated in a piece of soft silicon rubber substrate (Fig. 1a), which is sewn on the inner surface of the glove (Fig. 1b), with dimensions of 60 mm × 22 mm × 5 mm. The sensor is located over the hypothenar of the palm and the flexibility of its substrate allows it to adapt to different contours. The data processing package, with a size of 57 mm × 35 mm × 15 mm, is attached to the backside of the glove (Fig. 1c) and connected with the sensor through a wire. It is responsible for handling the signal from the sensor, supplying power, and transmitting data wirelessly. The glove is fabricated with a textile of good air permeability and suitable for long-time wearing. The total weight of the system is 86 g.

### Principle of measurement

The system employs a dual LED with two wavelengths—one (λ_1_ = 660 nm) is below the isosbestic point (λ_iso_ = 805 nm) and the other (λ_2_ = 905 nm) above. In this way, a considerable contrast can be achieved between oxygenated and non-oxygenated blood [11].

**Fig. 2:**
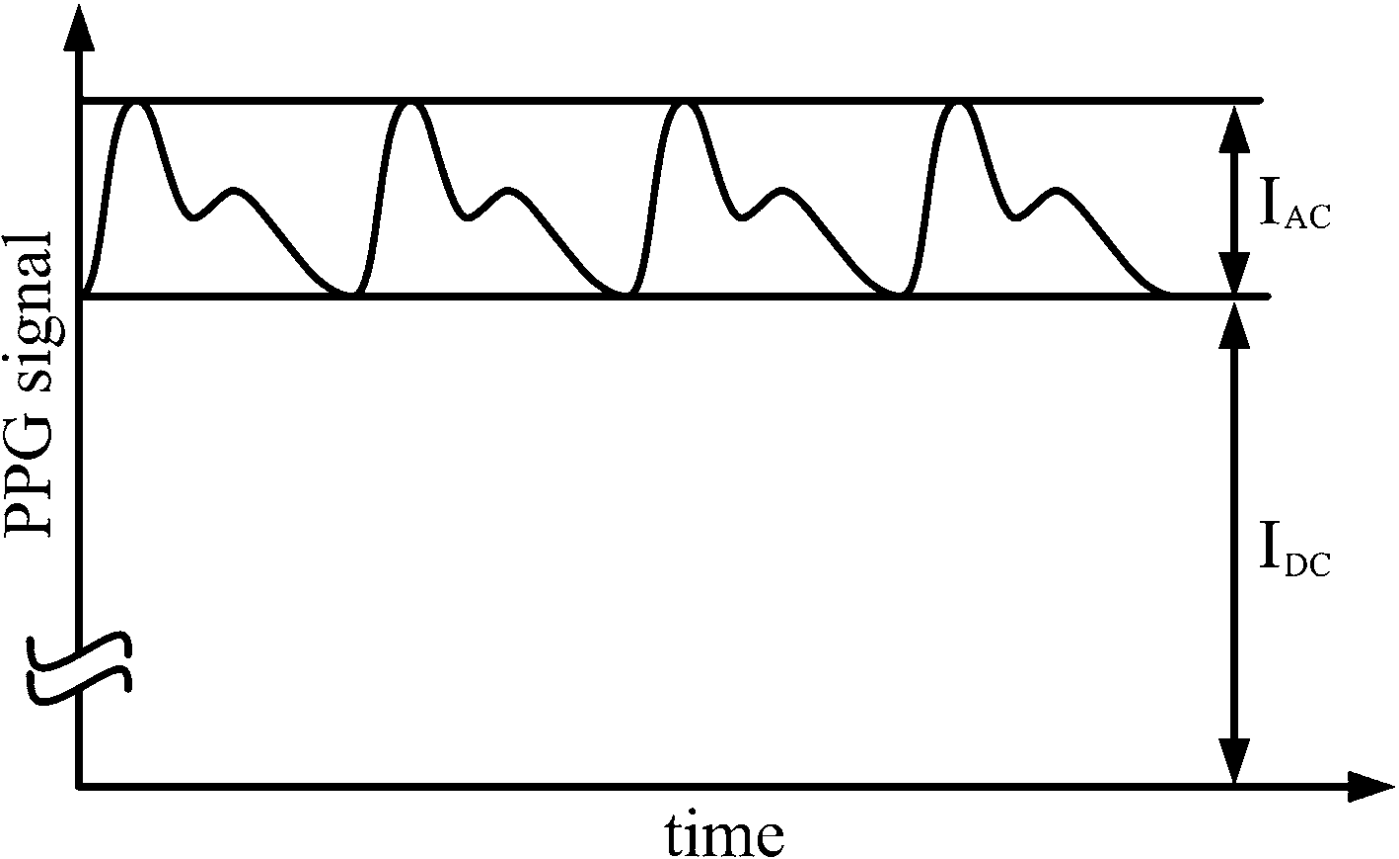
Characteristic PPG curve due to variations in dermal blood volume. I_AC_ represents the alternating component of the signal and I_DC_ the constant component.

As pulse waves propagate through the arteries, local dermal shifts in blood volume are induced by each heart contraction. Consequently, reflection and transmission of irradiated light change over time. This variation is called PPG pulse waveform, as shown schematically in Fig. 2. Most of the signal is static (DC) and represents the light that has not been modulated by pulsatile variation of arteries. This DC component is mainly caused by ambient light, direct light crosstalk between LED and PD, and the light reflected by tissues, venous blood and non-pulsatile arterial blood. A fairly small proportion of the signal is alternating component (AC), which results from the pulsation of arterial bed. The AC and DC components are both necessary for determination of SpO_2_ [12].

Taking into account the wavelengths used, the so-called R value (ratio-to-ratio) as introduced in [12] is calculated by:

1 $$R = \frac{{{{I_{{AC\_\lambda_{1} }} } \mathord{\left/ {\vphantom {{I_{{AC\_\lambda_{1} }} } {I_{{DC\_\lambda_{1} }} }}} \right. \kern-0pt} {I_{{DC\_\lambda_{1} }} }}}}{{{{I_{{AC\_\lambda_{2} }} } \mathord{\left/ {\vphantom {{I_{{AC\_\lambda_{2} }} } {I_{{DC\_\lambda_{2} }} }}} \right. \kern-0pt} {I_{{DC\_\lambda_{2} }} }}}}$$

and SpO_2_ is a function of R:

2 $$SpO_{2} = f(R)$$

In theory, the function about *R* can be supposed to be a linear function [12]. However, in practice, the relation between the R value and SpO_2_ must be acquired from “real” calibration measurements, because the theoretical hypotheses are relatively simple.

### Sensor design

**Fig. 3:**
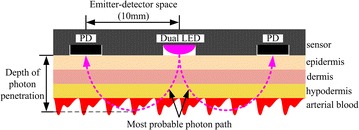
Structure of the sensor and photon migration. Photons ejected from LED get to PD through a parabolic trace.

The sensor contains a dual LED with wavelengths of λ_1_ = 660 nm (red) and λ_2_ = 905 nm (infrared), positioned at the center, and two PDs, arranged symmetrically on both sides of the LED (Fig. 3). This kind of multi-PD configuration can reduce the LED drive current and increase the signal quality [13]. As shown in Fig. 3, the most probable photon path from the LED to PD appears to be a parabolic line. The bottom of this line means the depth of photon penetration [14]. To a certain degree, a wider space between a LED and PD can result in a greater depth, which means more arterial blood can be detected to reinforce the signal strength [15]. According to [13–15], the LED/PD distance of the sensor was chosen to be 10 mm.

### Circuit

**Fig. 4:**
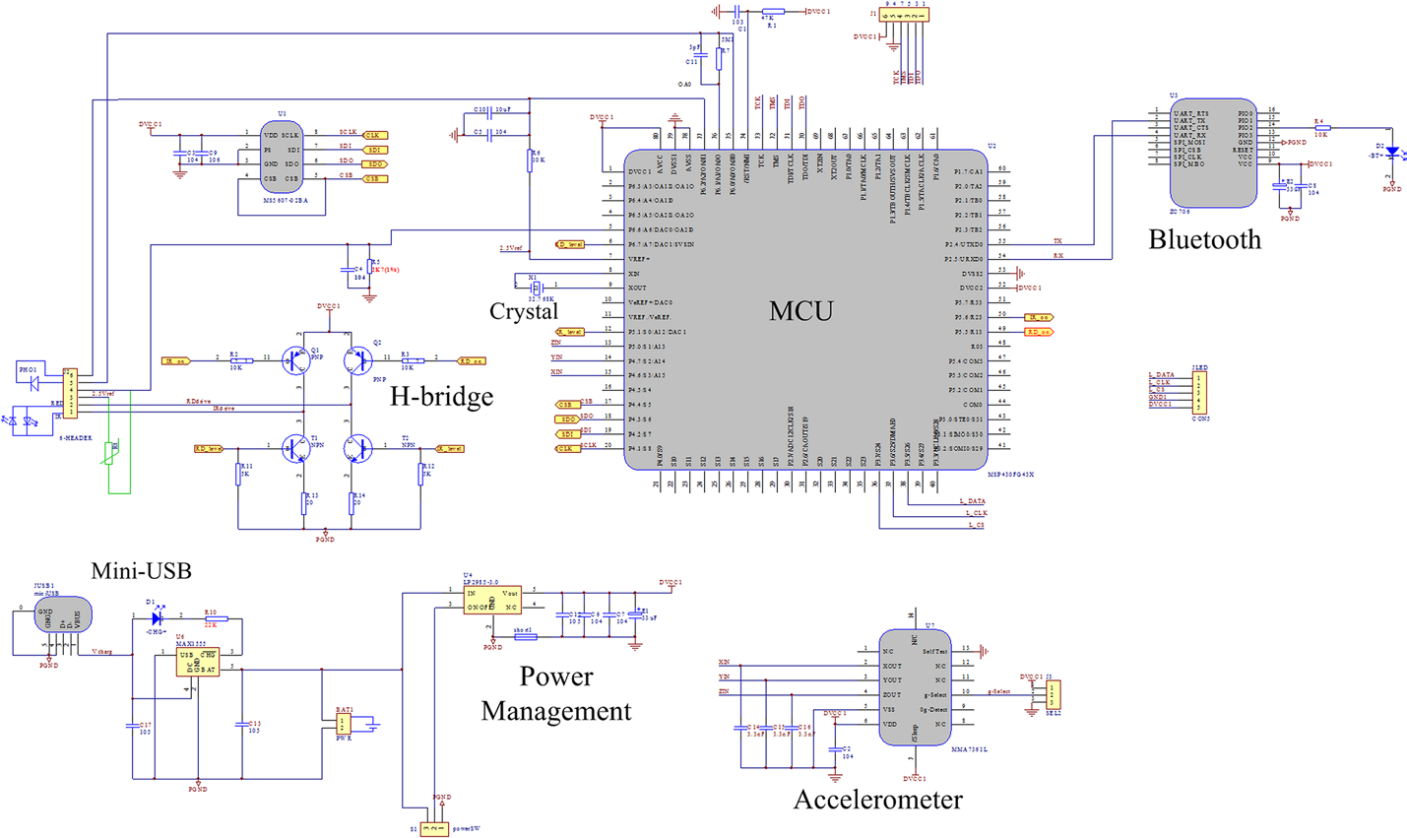
Circuit diagram of system. The system contains five main parts—LED driving unit (H-bridge), MCU, Bluetooth, power management and accelerometer.

The circuit diagram is shown in Fig. 4. The LED drive unit is an H-bridge mainly composed of two pairs of PNP (BC856A, ON Semiconductor) and NPN (MMBT2222, ON Semiconductor) transistors. It provides appropriate current to activate the red/infrared LED alternately according to the MCU (MSP430FG43X, Texas Instruments) instructions. The signal of PD is received and processed by the MCU. The wireless communication interface is a Bluetooth 4.0 module (ZC706, Zhichun Information Tech. Co., Nanjing, China), connected with the MCU through a RS232 serial port. A crystal of 32 kHz (DT-26, DASHINKU Co.) is applied as the timer of the MCU. The tri-axial accelerometer is MMA7361L (Freescale Semiconductor, Inc.), which is used to measure the acceleration signal representing motions. The system is powered by a 600 mAh Li-polymer battery, which can be recharged through the Mini-USB interface.

### Control flow of the system

**Fig. 5:**
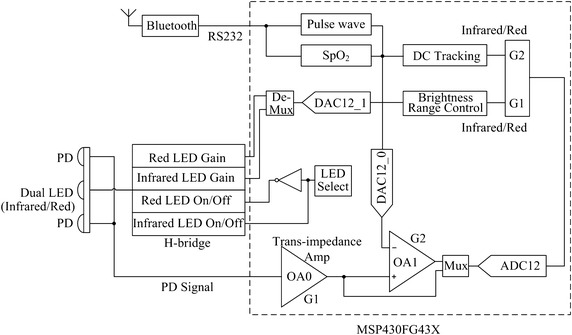
Control flow diagram. The red and infrared LEDs are activated alternatively. When each LED is on, the corresponding PD signal is sampled and the DC component in it is subtracted by DC tracking. The *left* red and infrared AC components are used to calculate SpO_2_.

**Fig. 6:**
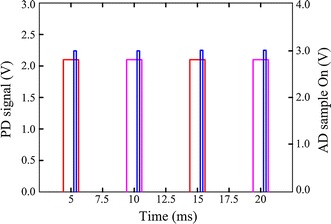
Timing and duty cycle of the LEDs. The *red* and *purple* pulses represent the PD signals when the red and infrared LEDs are switched on, respectively. The *blue* pulse indicates the activation of ADC.

Most processes of this system are undertaken by the MCU, including the regulating of LED driving current, the handling of PD signal, and the calculating of SpO_2_ (Fig. 5). The red and infrared LEDs are switched on/off alternately in accordance with a time sequence set by the timer of the MCU. They are turned on one after another at a cycle of 5 ms and their light-emitting periods are both 400 μs for a single cycle (Fig. 6). Therefore, the emitting proportion in a cycle, i.e., the duty cycle of each LED is just 8%, which means that 92% of the drive current will be saved. When the red or infrared LED is on, the PD is excited by the light source and generates a current from the received light.

The current is converted into a proportional voltage by the trans-impedance amplifier OA0. The output of OA0 is sampled by ADC12 with a sample rate of 200 Hz. As a result, the PD signal for each LED is sampled at 100 Hz (Fig. 6). Firstly, this raw signal is fed back to control the brightness of the LEDs such that the PD outputs corresponding to both LEDs match each other with a small tolerance. Because DC component makes up more than 98% of the total output strength (AC + DC) [16], the red and infrared DC components will be approximately equal to each other and can be neglected in the calculation of SpO_2_ (Eq. 1). Secondly, the raw signal is fed into a DC tracking unit using a digital tracking algorithm to pick out its DC component. This output is connected with the negative terminal of the amplifier OA1, while the raw signal connected with the positive one. As OA1 would only amplify the difference between its two terminals, the DC portion of the signal is subtracted and only the AC portion, i.e., the pulse wave is extracted and amplified. The pulse wave is sampled by ADC12 and utilized to compute SpO_2_ in the next step. At the same time, the acceleration signals at three orthogonal directions measured by the tri-axial accelerometer are sampled by another three ADC12 s. The processed data are transmitted to an upper computer via the Bluetooth module.

### In vivo calibration

Because the function of SpO_2_ about *R* value can not be precisely analyzed in theory, pulse oximeters have to be calibrated with the help of an in vivo experiment nowadays [17]. In that process, the pulse oximeter is applied to the participants and their arterial oxygen saturation (SaO_2_) is analyzed by blood-gas analysis (BGA). The participants’ blood oxygen levels are decreased by reducing the amount of their oxygen inhalation. The *R* value (Eq. 1) of the pulse oximeter and the corresponding SaO_2_ are recorded at the same time, and the relationship between them can then be determined. The human experiment also can be employed to examine the accuracy of a pulse oximeter by comparing its measurement (SpO_2_) to the SaO_2_.

We performed two human hypoxia experiments for the glove pulse oximeter at the People’s Liberation Army (PLA) General Hospital (Beijing, China). The first one calibrated the function of SpO_2_ about the R value and the second validated the accuracy. Twenty healthy participants (nonsmokers) were recruited for this study according to the recommendation of the International Standardization Organization [18]. The calibration group was comprised of three females and seven males ranging in age from 18 to 31 years old, while the validation group comprised of four females and six males from 20 to 28 years old. The study was approved by the hospital’s Ethics Committee and written informed consent was obtained from all participants.

**Fig. 7:**
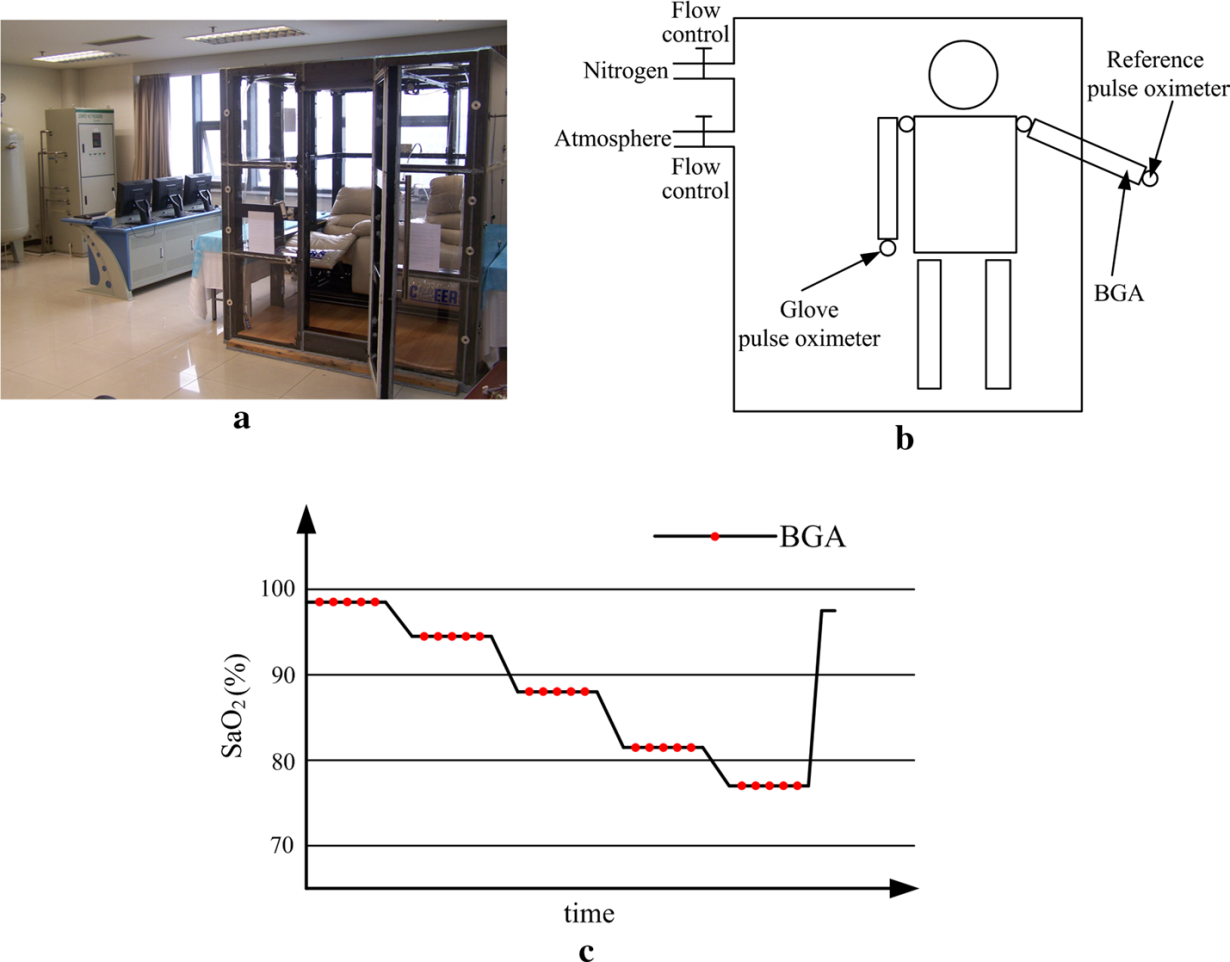
Hypoxia experiment design. **a** Cabin to make low oxygen conditions. **b** Design of the hypoxia study. **c** Protocol to obtain the relationship between the R value and the SaO_2_.

During the hypoxia experiments, the participant was lying motionless in a couch mounted inside an in-house made cabin (Fig. 7a), wearing the glove pulse oximeter on one hand. The SaO_2_ of the participant was regulated manually by changing the oxygen proportion in the air flowing into the cabin (Fig. 7b). The blood oxygen level was lowered in five steps from close to 100% down to 70–77% (Fig. 7c). A reference pulse oximeter (Radical 7, Masimo Co.) was applied to indicate which level the SaO_2_ of the subject had reached. After a saturation level was reached and stable for at least 30 s, five blood samples were taken from the radial artery in a time interval of 20 s with heparinized syringes (Preset Syringe 364314, BD Co.). The samples were analyzed by a BGA instrument (ABL80 FLEX CO-OX, RadioMeter Co.) and the results were used as the reference SaO_2_ values. Therefore, for each subject, 25 SaO_2_ samples were obtained and the total number of reference SaO_2_ values was 250. The BGA instrument was serviced and calibrated before the study.

By fitting the reference SaO_2_s on the corresponding R values, the function of SpO_2_ about R of the system was calibrated and then implemented in the firmware. After calibrated, the system was employed to measure the SpO_2_s of the participants in the following validation experiment, while the SaO_2_s were also sampled at the same time. The relationship between the SpO_2_ and SaO_2_ values (n = 250) was analyzed by Pearson correlation and linear regression. The accuracy was calculated based on the deviation between them.

### Accelerometer-based adaptive motion artifact cancellation

PPG signals are susceptible to motion interferences, leading to damaged accuracy of SpO_2_ measurement. The most promising approach that can be realized in real-time to reduce motion artifacts is adaptive noise cancellation (ANC) [19]. The method utilizes acceleration signals as a reference to the motion artifact components present in the corrupted PPG signals.

**Fig. 8:**
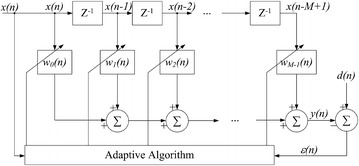
Block diagram of adaptive filter. The reference noise *x(n)* is passed through a delay line (represented by Z blocks). The tap-weights (*w*_i_ *(n)*) multiply the delayed *x(n* − *i)*, which are summed to form *y(n)*. Because *y(n)* approaches the true noise in *d(n)*, the difference between them *ε(n)* form the output with the noise cancelled. The adaptive algorithm (LMS or RLS) regulates *w*_*i*_ *(n)* based on *ε(n)*.

The core of ANC is an adaptive filter, which is a type of filter that provides adjustable cut-off frequency based on filter input and output. This type of filter is suitable for certain applications in which the noise frequency to be removed, such as motion artifact, is not known a priori. An adaptive filter block diagram is illustrated in Fig. 8. The adaptive filtering process consists of three separate stages. First, the noise reference input signal (acceleration signal) ***x***(n) = [*x(n), x(n* − *1), x(n* − *2),…, x(n* − *M* + *1)*]^*T*^ is filtered, where *M* is the filter order. Second, the filtered noise reference output *y(n)* is subtracted from the desired signal *d(n)*, which is the corrupted PPG signal by motion. Third, the filtering coefficients, or tap-weights ***w****(n)* = [*w*_*0*_*(n)*, *w*_*1*_*(n)*, *w*_*2*_*(n)*,*…*, *w*_*M* − *1*_*(n)*]^*T*^ are adjusted based on the difference between *d(n)* and *y(n)*, i.e., the error *ε(n)*. Because the acceleration is correlated with the motion artifact in PPG [20], *y(n)* will approach the motion noise in *d(n)* after a few iterations and the difference between them *ε(n)* will approximate the restored PPG signal with the motion artifact canceled. The filter tap-weight vector ***w****(n)* is calculated iteratively based on an updating algorithm such as the least mean squares (LMS) and recursive least squares (RLS).

A primary advantage of the LMS algorithm is that a lower number of computations are required relative to RLS. However, RLS algorithm provides a faster learning rate and can obtain a smaller error at the cost of longer execution time [21]. To compare these two algorithms, they were implemented offline in Matlab to process the corrupted PPG data. The signals from the accelerometer (Fig. 4) at three orthogonal axes were summed and filtered by a 6th order Butterworth band-pass filter (f_c1_ = 0.5 Hz, f_c2_ = 5.0 Hz) to obtain the AC component. This signal was provided as the reference noise signal ***x****(n)* to the LMS and RLS ANC, while the corrupted PPG signal given as the desired signal *d(n)*. The filter tap-weight vector ***w****(n)* of both algorithms were initialized to 0. The initial inverse covariance matrix ***P***(0) of the RLS was set as 0.1***I***, where ***I*** is an identity matrix. The step-size *μ* of the LMS algorithm and the forgetting factor *τ* of the RLS algorithm were chosen to be 0.016 and 0.99, respectively. Thirty-seven segments of corrupted PPG signals taken from the validation experiment were processed by the two algorithms. For each segment, the SpO_2_ root mean square error (RMSE) was quantified based on the differences between the processed results and the measurements of the reference Masimo pulse oximeter. Then the mean and stand deviation of the RMSEs for all segments were calculated to evaluate the performance of motion artifact cancellation. The efficiencies in motion resistance of these two algorithms with different filter order *M* were evaluated by the trade-off between their performance and complexity of computation.

## Results

### In vivo calibration and evaluation of accuracy

**Fig. 9:**
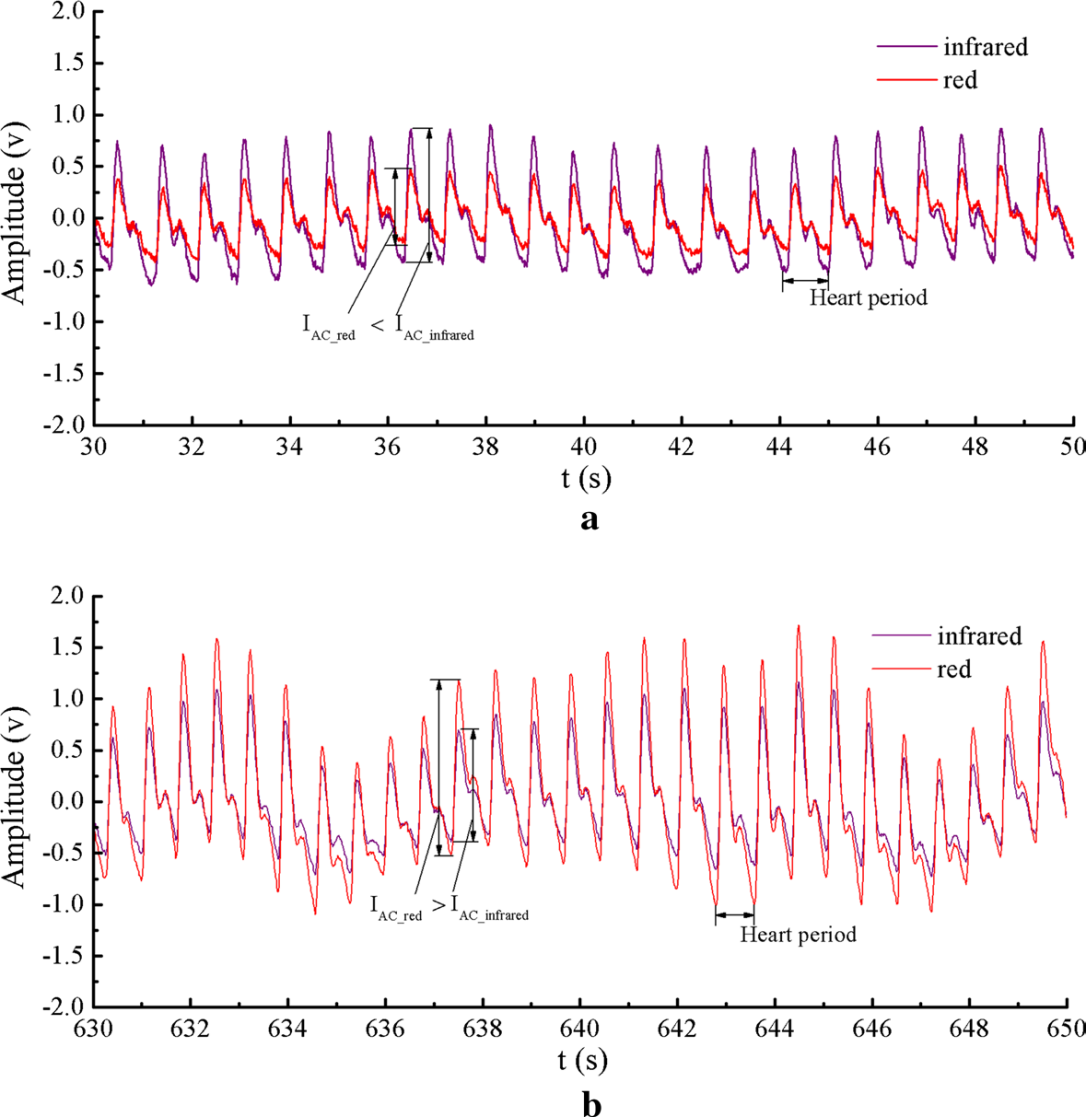
Two periods of pulse wave. **a** Red and infrared pulse waves when SaO_2_ is 98%. **b** SaO_2_ is 72%.

Figure 9 shows two periods (20 s) of pulse wave signals of one participant measured at high (98%) and low (72%) SaO_2_. When the oxygen saturation level was decreased, the signal exhibited larger fluctuations due to deeper breath and the heart period became shortened, which were induced by the physical protection mechanism against hypoxia. The ratio of the red AC amplitude to the infrared one (i.e., the R value) was less than 1 at high SaO_2_, and increased to larger than 1 at low SaO_2_. The results demonstrated that PPG signals were properly measured by the system for normal range of SaO_2_.

**Fig. 10:**
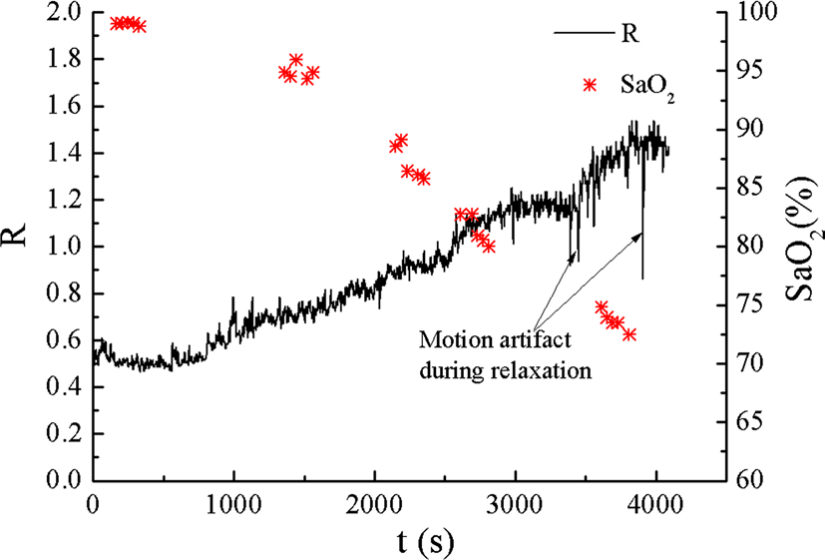
R curve of one subject in a calibration experiment. For one subject, SaO_2_ was sampled 25 times for the full range while the R curve was recorded simultaneously.

Figure 10 presents the complete R curve of the participant and the BGA associated with it. The R value of the system showed a good correlation (negative) with the SaO_2_. The large interference happened in the curve was caused by the hand motion of the participant, who was allowed to relax during the idle period when a stable blood oxygen level had not been reached.

**Fig. 11:**
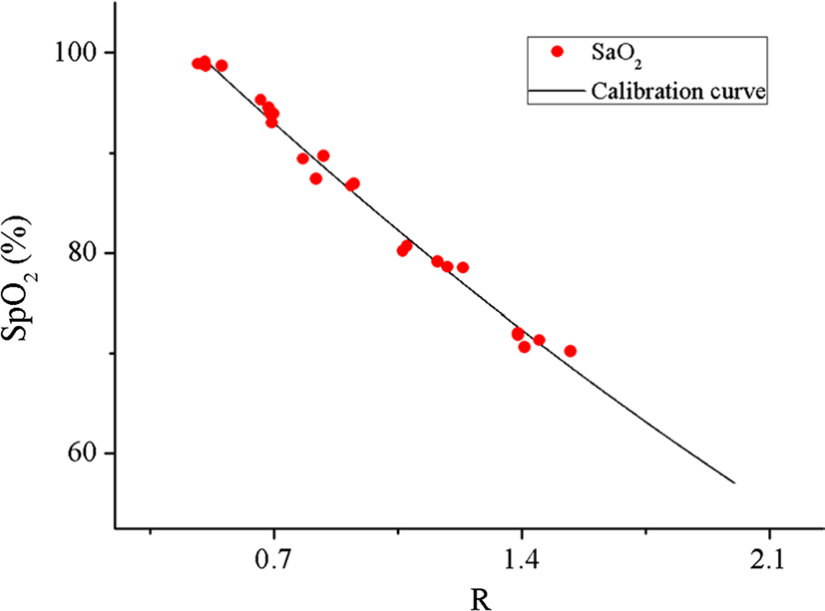
An individual calibration curve of SpO_2_ on the R values. For this subject, the function of SpO_2_ about R was got by fit the reference SaO_2_ on R using second-order polynomial (RMSE = 1.07%, *ρ* ^2^ = 0.99).

Figure 11 shows an individual calibration curve of SpO_2_ on R. A second-order polynomial was used for calibration, as mentioned in [22]:

3 $$SpO_{2} = A \times R^{2} + B \times R + C$$

**Fig. 12:**
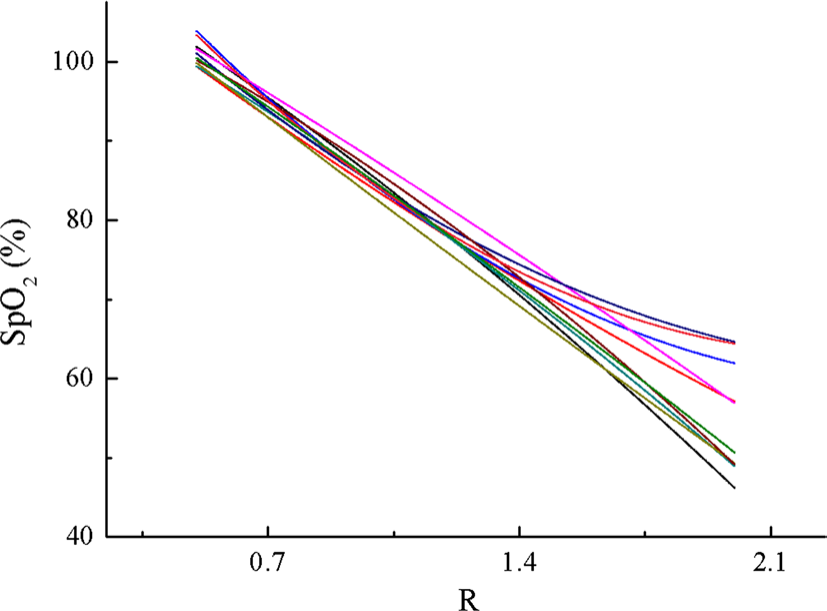
Individual calibration curves of all the participants in the calibration experiment. All the subjects’ calibration curves were consistent with each other for the full SaO_2_ range, except less than 70%.

After calibrated, the RMSE between the SpO_2_ and SaO_2_ was 1.06%, with a strong correlation (determination coefficient *ρ*^2^ = 0.99). Individual curves of all the participants in the calibration experiment are shown in Fig. 12. These curves are consistent with each other for the full range of SpO_2_. However, when SpO_2_ was less than 70%, the deviation between them became larger. This is caused by two reasons: one is the accuracy of SpO_2_ measurement varied at low level and the other is the samples of reference SaO_2_ values lower than 70% were not sufficient, because this hypoxia level was rarely reached to prevent harm to the subjects. Table 1 shows the coefficients of the curves’ functions according to Eq. (3). These polynomials differ from each other, since physiological differences at the measuring site exist between individuals. However, most of these functions have a considerable accuracy (<2.5%) and strong correlation (>0.95), except no. 7. The mean RMSE and *ρ*^2^ of all the fits are 1.81% and 0.966, which indicate it is valid to apply quadratic polynomial to fit. The max deviation among the curves is less than 10% (Fig. 12). Therefore, it is possible to obtain a general function based on all the measurements utilizing quadratic polynomial.

**Table 1:** Coefficients of all individual calibration curves

Subject	*A*	*B*	*C*	*ρ* ^*2*^	*RMSE*
1	−3.84	−27.55	116.6	0.986	1.365
2	3.10	−36.00	116.7	0.990	1.065
3	11.29	−56.26	129.2	0.988	1.221
4	3.65	−24.58	112.7	0.977	1.431
5	−1.51	26.07	115.0	0.964	2.074
6	0.69	−35.47	117.5	0.953	2.421
7	8.94	−46.67	122.2	0.879	3.419
8	−5.96	−19.18	111.3	0.979	1.509
9	12.14	−56.37	128.6	0.980	1.371
10	−1.69	−29.03	115.4	0.965	2.218
Mean				0.966	1.809

*A*, *B* and *C* coefficients of the fit function according to Eq. (3), *ρ* ^*2*^ determination coefficient of fit, *RMSE* root mean square error of fit.

**Fig. 13:**
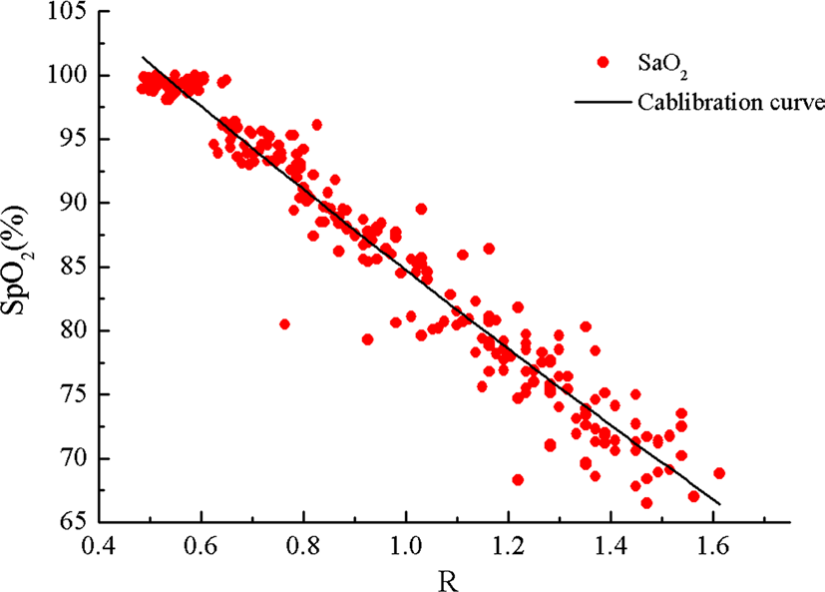
Curve of SpO_2_ on the R values of the calibration experiment. All the 250 SaO_2_ and R values were utilized to fit (RMSE = 2.27%, *ρ* ^2^ = 0.95).

In order to get a good repeatability, a representative curve must be gained on the calibration points of all the participants in the first experiment, as shown in Fig. 13. The coefficients were 2.23, −35.65 and 118.1 for A, B and C (Eq. 3), respectively, and the RMSE was 2.27% with *ρ*^2^ equal to 0.95.

**Fig. 14:**
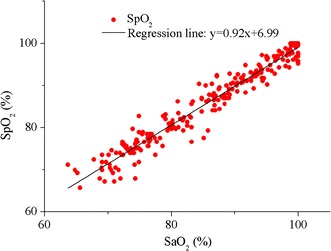
Correlation and regression line of SpO_2_ with SaO_2_ in the calibration experiment. The Pearson correlation coefficient of SpO_2_ with SaO_2_ was 0.97.

**Fig. 15:**
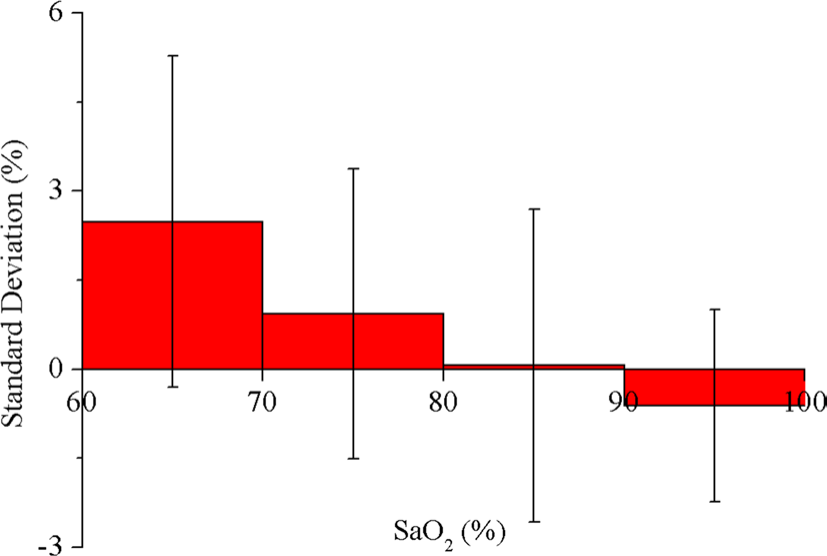
Errors between SpO_2_ and SaO_2_ for different intervals covering 60–100%. Each column’s width means an interval of 10% SaO_2_. *Error bars* indicate ±1 SD of the deviations for corresponding intervals.

**Fig. 16:**
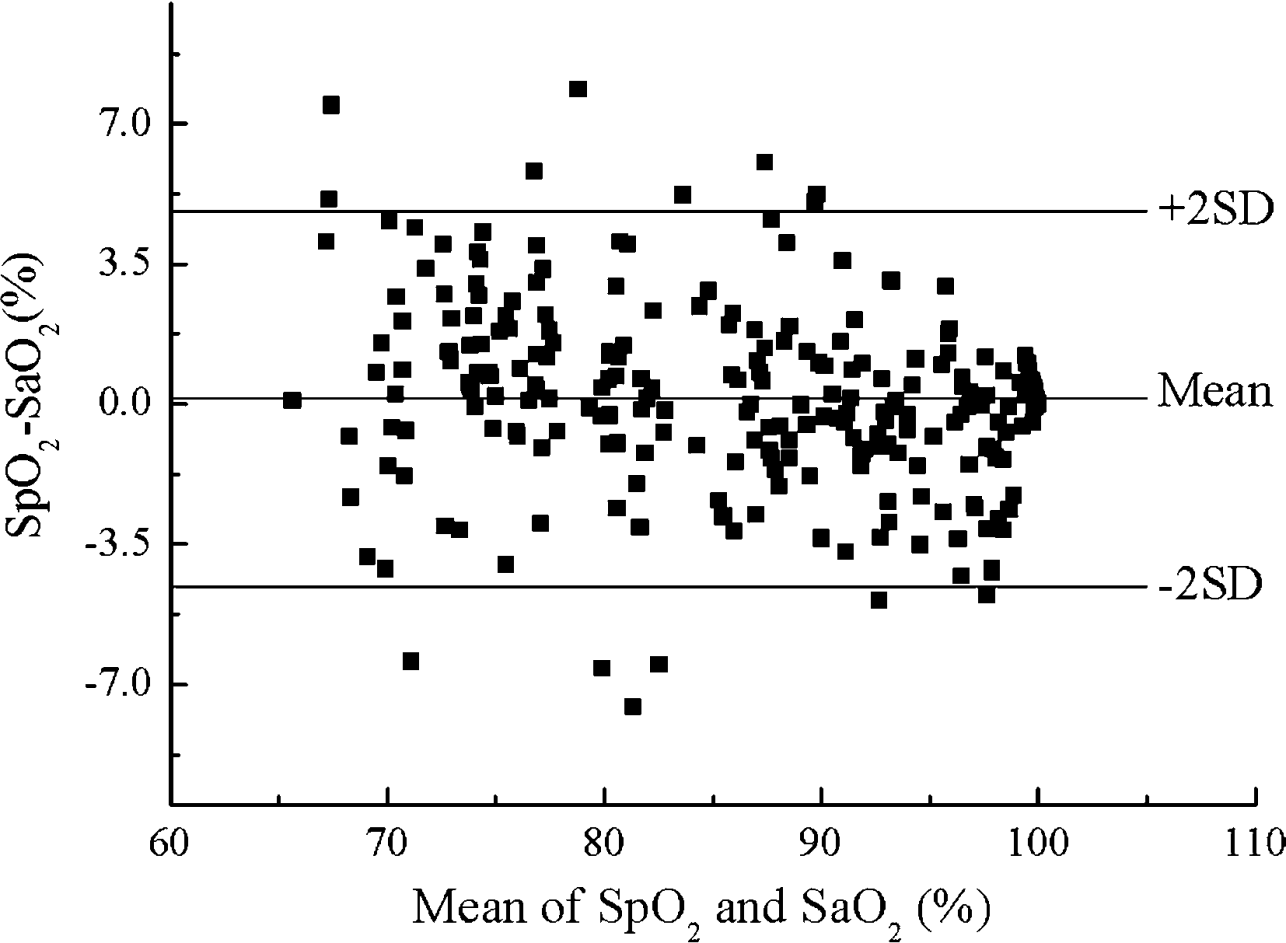
Bland–Altman graph of SpO_2_ versus SaO_2_ of the validation experiment. The *dots* represent all the deviations. The *middle solid line* represents the mean of deviations, while the *upper* and *under lines* indicate +2SD and −2SD, respectively.

After calibrated by the first experiment, the function of SpO_2_ about R was implemented in the system’s firmware. Then the system was used to measure SpO_2_ in the validation experiment and its results were compared to the simultaneously sampled SaO_2_s. There were 250 data pairs obtained in this experiment. In Fig. 14, the SpO_2_ values were plotted against corresponding reference SaO_2_ measurements and they showed a strong correlation (Pearson correlation coefficient = 0.97). The slope and intercept of the regression line between them were 0.92 and 6.99, respectively. The error was defined as SpO_2_ minus SaO_2_. Firstly, the mean values and standard deviations (SD) of the errors were calculated for four equal SaO_2_ intervals between 60 to 100%. As shown in Fig. 15, the error was largest (2.48 ± 2.79%) when SaO_2_ was lower than 70%. With the increase in SaO_2_, the error decreased and the least one was −0.62 ± 1.62% when SaO_2_ was higher than 90%. For the full range of SaO_2_, the mean error was 0.12% with an SD equal to 2.34%, as shown in Fig. 16. The errors spread uniformly on the normal range of SpO_2_ and 94.4% of the total measurements lies in the range of mean ± 2SD. The accuracy of the system is also 2.34%, calculated according to the definition for accuracy of a pulse oximeter in [18]:

4 $$Accuracy = \sqrt {\frac{{\sum\limits_{i = 1}^{n} {\left( {SpO_{{2_{i} }} - SaO_{{2_{i} }} } \right)^{2} } }}{n}}$$

where n is 250, the number of SaO_2_ samples.

**Fig. 17:**
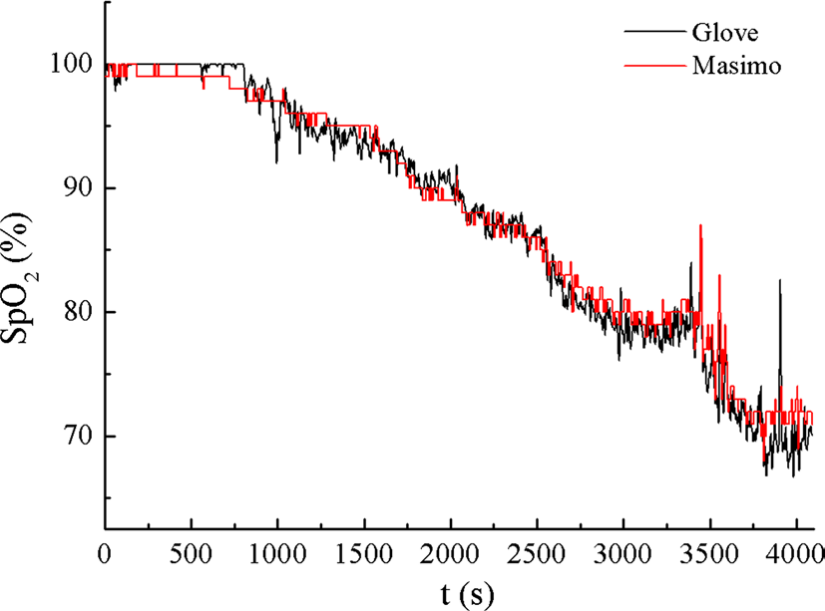
Comparison to the reference Masimo equipment. A complete comparision between the system’s and Masimo’s measurement traces for one subject in the validation experiment.

Because the system’s accuracy has been validated by comparing to SaO_2_, it is not necessary to analyze the error between the system and Masimo once more. However, Fig. 17 shows a comparison between them and the significant consistency demonstrates that the glove pulse oximeter can continuously monitor SpO_2_ properly. In all the experiments, it has been worn for at least 1 h by each of the twenty participants. The degree of wearing comfort was accepted by all the participants.

### Motion artifact cancellation

**Fig. 18:**
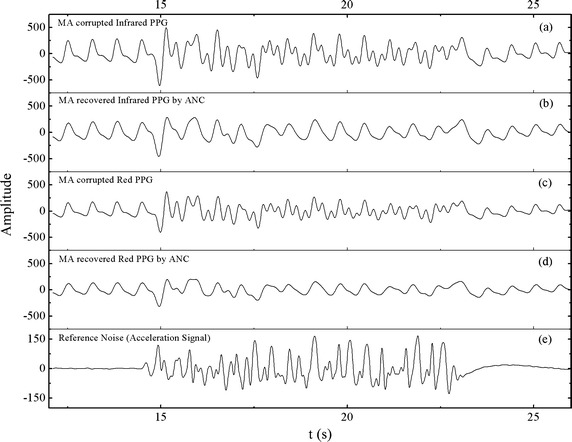
Representative raw PPG, adaptively filtered PPG and hand acceleration signals. **a** Typical infrared PPG signals before and **b** after processing by the ANC algorithm (LMS, *M* = 24). **c** Typical red PPG signal before and **d** after processing by the ANC algorithm (LMS, *M* = 24). **e** The corresponding reference noise obtained simultaneously from the accelerometer.

Representative hand acceleration, raw PPG signals acquired during motion and the adaptively filtered PPG signals are shown in Fig. 18. The adaptively filtered PPG signals (Fig. 18b, d) show that PPG peaks can be more easily identified than raw signals (Fig. 18a, c). For example, several portions of the adaptively filtered infrared signal appear to be more typical, clean PPG signals (Fig. 18b, t = 17, 18, 20, 21 s).

**Fig. 19:**
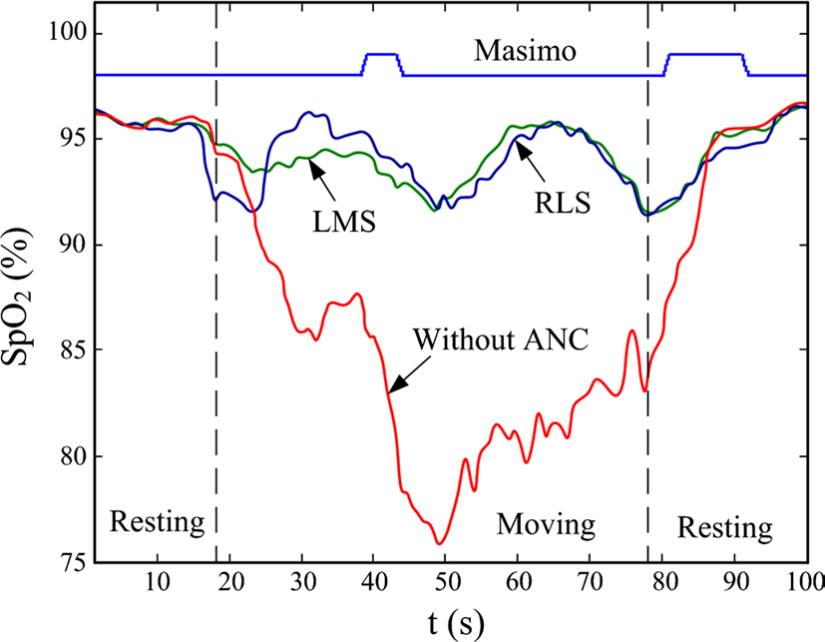
SpO_2_ measurements processed by ANC compared to Masimo. The filter order M of LMS and RLS are both 24.

**Fig. 20:**
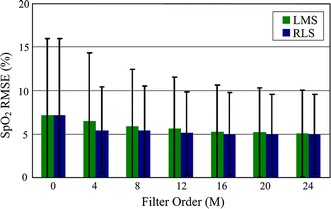
SpO_2_ RMSEs for different filter orders. *Error bars* indicate ±1 SD of RMSEs for 37 segments’ errors between the glove pulse oximeter and Masimo measurements processed with varying filter orders *M*. *M* = 0 means the error obtained without ANC.

Figure 19 shows a representative tracing of SpO_2_ measurements obtained from the custom pulse oximeter with and without ANC. Reference measurements were obtained simultaneously from the Masimo pulse oximeter. The motion interferences were suppressed when ANC was applied. A total of 37 segments of corrupted PPG signals from the validation experiment were processed by the LMS and RLS algorithms with different filter order *M*. The mean and standard deviation of the RMSEs observed from all segments are summarized in Fig. 20. Analysis of the data revealed that utilizing either the LMS or RLS algorithm to process the corrupted PPG signals can improve SpO_2_ accuracy during motion. Figure 20 also shows that the performances of both algorithms depend on the filter order used to implement each algorithm.

### Power consumption

**Fig. 21:**
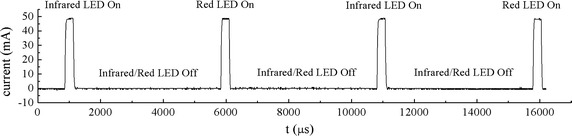
Drive current of the LEDs measured at work. Red and infrared LEDs were turned on alternately to save power.

The drive current of the LEDs at work was measured, as shown in Fig. 21. Each LED’s light-emitting time was 396 μs and idle time 4,576 μs. The peak drive current was 49 mA and the mean current was about 4 mA, because the duty cycle was 7.96%. The total power consumption of the system was evaluated by measuring the output current of the battery. When the Bluetooth was switched off, the total current was 6 mA. It increased to 8 mA when the Bluetooth began to transmit data.

## Discussion

A novel pulse oximetry system in form of a glove has been developed and clinically tested in a human hypoxia study. In contrast to classical transmissive mode measurement devices, the new system uses a reflective mode realizing measurement at hypothenar. This system was light-weighted and easy to wear. The feasibility of SpO_2_ measurement with the system has been demonstrated in two human hypoxia experiments (n = 20). First, the function of SpO_2_ on R value was calibrated using a second-order polynomial for 10 healthy participants in the calibration experiment. Because the PPG signal was properly detected at all hypoxia levels, each individual function was acquired with a small RMSE and strong correlation, except no 7. This was mainly because the glove’s size was not suitable for the subject’s hand and the sensor was not attached at hypothenar very well. If a series of gloves with different sizes were manufactured, the sensor would be guaranteed to fit different individuals and the calibrated accuracy would be improved further. Nevertheless, the deviation between those calibration functions was not significant and thus we can achieve a general calibrated function on all the points got in the calibration experiment. Second, this function was implemented in MCU and the accuracy was verified in the validation experiment for another 10 healthy participants. Because the SaO_2_ samples lower than 70% were difficult to be obtained in the calibration experiment, the function for this range was not calibrated precisely. Hence, when SaO_2_ was lower than 70%, the proved accuracy was not favorable. However, because the SaO_2_ samples higher than 70% accounted for the majority of the total ones and the accuracy for this range was relatively high, the system’s global accuracy was 2.34% for the full normal measurement range. In addition, the accuracy proved in the validation experiment was consistent with the RMSE (2.27%) of the general function obtained in the calibration experiment. This indicates that the custom pulse oximeter can achieve a repeatable SpO_2_ measurement with a good accuracy for different individuals and SpO_2_ levels. The accuracy of the system is acceptable according to the defined level in [18] (<4%). However, in addition to individual physiological differences (e.g., different thickness of each layer of skin), the light crosstalk between the LED and PD of the sensor may lead to the deviation of measurement among individuals. If methods to avoid this crosstalk, such as set a barrier, were applied, the accuracy of the system would be increased.

The total current needed at normal work mode was 8 mA. Since the capacity of the battery is 600 mAh, the system is able to work for at least 60 h on a single charge. To improve the performance of the system during motion, we investigated the effectiveness of the LMS and RLS ANC algorithms with a tri-axial accelerometer as the noise reference input. Analysis of the data processed by them showed that ANC implemented using the LMS and RLS algorithms can help to improve the accuracy, as shown in Fig. 17. We also found that the degree of improvement depends on the filter order *M* used to implement each adaptive algorithm (Fig. 18). The trade-off between computation complexity and performance is important since our goal is to implement ANC to resist motion artifact in real-time. For example, an implementation based on a 24th order filter would provide an acceptable error reduction, which implies that the LMS algorithm will require only 24 operations compared to 576 operations that will be required by an RLS algorithm. The motion-resistant effects of both algorithms were limited and especially damaged when the frequency and amplitude of movements varied in a dramatically stochastic way. This could be caused mainly by the unstable performances of both algorithms for treating various MAs with fixed parameters, i.e., the step size and forgetting factor. For better motion-resistant performance, deeper work should be done to study the effects on PPG imposed by different kinds of movements, such as translational and rotational motions, in addition with the algorithms’ parameters corresponding to these movements.

The glove pulse oximeter measures SpO_2_ at the hypothenar of hand and has to face a wide range of motion interferences, although some methods have been adopted to suppress MAs. However, the glove form can ensure a reliable sensor attachment with least discomfort for long-time monitoring and we think the system may be applicable for sleep monitoring.

## Conclusions

We have developed a new pulse oximetry system measuring PPG and SpO_2_ at hypothenar based on a reflective sensor. The accuracy and repeatability of SpO_2_ measurement with this system has been proved in the human hypoxia experiment. With small size and low weight, the system was integrated into a glove for easy wearing. Furthermore, it can work for a long time in wireless communication mode. We applied the LMS and RLS adaptive algorithms with the acceleration signal as the reference noise to suppress the motion artifact. Although more work should be done to improve the performance for resisting motion interference in real-time, this novel system promises to be a wearable wireless solution for healthcare monitoring.
